# In-person prospective audit and feedback on an oncology ward: development of an immunocompromised antimicrobial stewardship program

**DOI:** 10.1017/ash.2024.446

**Published:** 2024-10-15

**Authors:** Hannah Imlay, Sage B. Greenlee, Brandon J. Tritle, Nora F. Fino, Emily S. Spivak

**Affiliations:** 1 Division of Infectious Diseases, Department of Internal Medicine, University of Utah Health, Salt Lake City, UT, USA; 2 Department of Pharmacy, University of Utah Health, Salt Lake City, UT, USA; 3 Department of Pharmacy, Henry Ford Macomb Hospital, Clinton Twp., MI, USA; 4 Division of Epidemiology, Department of Internal Medicine, University of Utah, Salt Lake City, UT, USA

## Abstract

**Objective:**

To describe clinical syndromes, opportunities for antimicrobial optimization, and acceptance of recommendations made by an immunocompromised antimicrobial stewardship program performing in-person prospective audit and feedback (IPPAF) on inpatient oncology services.

**Design:**

Retrospective cohort study.

**Setting:**

Three inpatient oncology services including patients with solid tumor malignancies in an academic cancer center.

**Patients:**

Hospitalized adults with oncologic malignancies receive antimicrobials for any indication.

**Methods:**

We reviewed all patients receiving antimicrobials on inpatient oncology services who were included in IPPAF and prospectively documented clinical syndromes represented, most common recommendations, and acceptance rate. We also examined the standardized antimicrobial administration ratio (SAAR) for oncology units over the study period.

**Results:**

Over 34 weeks, we performed 154 interventions for 138 patients. Metastatic malignancy was common (52%) and 90-day mortality was high (43%). Diagnostic uncertainty was common (33/154, 21%), as were cases of intra-abdominal pathology (30/154, 19%), pneumonia (25/154, 16%), and urinary tract infection (12/154, 8%). The most common recommendations were changes in duration (63/154, 41%) and stopping antimicrobials for syndromes determined to be noninfectious (29/154, 19%). Acceptance of interventions was high (77% overall) and several SAARs on the primary oncology unit significantly decreased after starting IPPAF.

**Conclusions:**

We identified numerous opportunities for antimicrobial optimization among solid tumor malignancy patients. Most clinical syndromes were ones also encountered frequently in non-oncology populations, but several were unique and represented opportunities for targeted education.

## Introduction

Antimicrobial stewardship programs (ASPs) are mandated by the Centers for Medicare and Medicaid Services and improve antimicrobial use (AU) and clinical outcomes.^1^ Implementation of ASPs specific to immunocompromised patients has gained attention and momentum^2–4^ due to a high risk of multi-drug resistant organisms, adverse antimicrobial effects, and *Clostridioides difficile* infections among immunocompromised patients.^5^

Despite opportunities for optimizing AU,^6,7^ studies that characterize the implementation and impact of immunocompromised ASP interventions are limited. In part, this is due to the perceived risk of harm associated with withholding or decreasing AU in this complicated population.^6,8^ In-person prospective audit and feedback (IPPAF), or “handshake stewardship,” has been emphasized as a stewardship strategy among immunocompromised patients to enhance visibility of ASPs and gain buy-in from clinicians.^9^

In this study, we describe our experience developing an immunocompromised ASP and performing IPPAF on an inpatient oncology service. Our aims were to identify clinical syndromes unique to this population and demonstrate the feasibility of an IPPAF program by examining the type of ASP interventions recommended, the number of days that IPPAF occurred, the acceptance rate, and how unit-level AU changed over time.

## Methods

### Study setting

We reviewed patients with solid tumor malignancies who were receiving antimicrobials while admitted to Huntsman Cancer Hospital, a 148-bed free-standing cancer hospital affiliated with the University of Utah.

During the study period, patients with solid tumor malignancies or primary central nervous system lymphoma were cared for by three teams: (1) a hospitalist-only service (“hospitalist team”), (2) a team of internal medicine residents and a hospitalist attending (“teaching team”), or (3) a team of advanced practice clinicians (APCs) and an oncology-trained attending (“APC team”). Patients were primarily roomed on an oncology ward but occasionally were located on other wards. Our immunocompromised ASP is made up of one physician with a clinical focus on immunocompromised infectious diseases [0.3 full-time equivalent (FTE) specifically for stewardship in immunocompromised patients] and two ID-trained pharmacists (1.0 FTE dedicated for stewardship combined). This work was pursued as part of quality improvement and did not require institutional review board oversight.

## Intervention

All patients admitted to medical oncology services on antimicrobials were reviewed in preparation for IPPAF co-led by the ASP pharmacist and physician every Monday/Wednesday/Friday. In addition, patients with positive blood cultures were discussed on all weekdays. Patients were excluded if they were being managed through infectious disease consultation. IPPAF occurred from 1/1/2023 to 12/15/2023 for all patients on the oncology services receiving antimicrobials; however, we prospectively documented our interventions in detail from 4/24/2023 to 12/15/2023 so considered this time frame our study period.

IPPAF discussions held with each oncology team focused on six major questions loosely based on the 4 moments of antimicrobial prescribing^10^: (1) Does the patient have a clinical syndrome that requires antimicrobials? (2) Is the diagnosis clear? (3) Is antimicrobial choice appropriate? (4) Is antimicrobial duration appropriate? (5) Can intravenous antimicrobials be switched to oral antimicrobials?, and (6) Would the patient benefit from infectious diseases consultation due to the complexity or lack of clarity of their clinical picture?. Patients with oncologic malignancies or other immunocompromising conditions are frequently excluded from clinical guidelines^5^ but are included in some clinical trials assessing antimicrobial spectrum and duration. Antimicrobial therapy recommendations were supported by existing guidelines and literature. When there were insufficient data among immunocompromised patients, recommendations were extrapolated from data among immunocompetent patients (e.g., for patients with uncomplicated Gram-negative bacteremia, we recommended 7 days of therapy and switching from intravenous to oral antimicrobial therapy after patients stabilized). Infectious disease consultation was recommended among patients with complicated infections (i.e. undrained source of infection), patients who were not improving, or patients whose clinical picture was unclear.

Rounds also frequently included impromptu teaching opportunities. Among patients for whom an intervention was suggested, progress notes (under an “antimicrobial stewardship” service) were frequently left in patients’ medical charts highlighting the recommendations and rationale (Supplemental figure 1). The decision to place notes was at the discretion of the ASP team but was largely based on complexity of case, primary team/pharmacist request, and familiarity of teams with IPPAF (e.g., more notes were left earlier in the study period).

### Study outcomes

The primary aims of our study were to identify ASP opportunities in this population and describe the feasibility of IPPAF. To identify ASP opportunities in this population, we described the demographics and clinical syndromes of patients on the day of IPPAF as well as recommended interventions. Patients were included in the dataset multiple times if they had distinct recommendations (including distinct recommendations given on the same day or given within the same admission encounter on different days as clinical course progressed). We described clinical syndromes, number of notes left in the patient chart, and recommendations from in-person discussions.

To examine feasibility of program structure, we identified the number of days (out of all Mondays/Wednesdays/Fridays within the study period) that IPPAF occurred. IPPAF was not performed on days when either the ASP pharmacist or physician had service responsibilities, vacations, or other conflicting commitments. IPPAF was defined as days in-person rounding occurred with both the pharmacist and physician present; “hybrid PAF” was defined as days only one of the ASP team members rounded in person or days when recommendations were given via telephone or secure chat.

Our institution submits AU data to the National Healthcare Safety Network. To examine ASP impact, we evaluated the standardized antimicrobial administration ratios (SAARs) of oncology wards and intervention acceptance. Our oncology SAARs were derived from a hospital unit that primarily included patients with solid tumor oncology but also included hematology, and stem cell transplant patients. We evaluated SAAR levels before and after the implementation of IPPAF using interrupted time series (ITS) models. Each type of SAAR (Broad spectrum antimicrobial agents predominantly used for hospital-onset infections [BSHO], Broad spectrum antibacterial agents predominantly used for community-acquired infections [BSCA], Antibacterial agents predominantly used for resistant Gram-positive infections [Gram positive], Narrow-spectrum beta lactam agents [NSBL], Antibacterial agents posing highest risk for *C. difficile* infection [CDI], Antifungal agents predominantly used for invasive candidiasis [Antifungal]) was modeled in a separate linear regression model based on monthly SAARs over time, allowing for a change in slope after the intervention had begun. We did not test for an immediate effect of the intervention, as we expected that effects of the intervention would occur over time. We assessed autocorrelation using Durbin-Watson tests and residual plots. We used the ITS models to estimate test for differences in the pre- and post-intervention slopes for monthly changes in SAARs.

## Results

Over 34 weeks of IPPAF, the stewardship team performed 154 interventions for 138 patients. Demographics of patients are summarized in Table 1. Notably, patients had a high rate of metastatic disease at presentation (52%) and mortality (43% 90-day mortality from date of IPPAF intervention).

**Table 1.** Demographics of the cohort

Demographics	Patients n = 138 (%)
Male	67 (48)
Age, median (IQR)	64 (53–71)
**Type of malignancy**	
Lung^a^	17 (12)
Breast	15 (11)
Head/Neck SCC	14 (10)
Colon/rectal	12 (9)
Pancreatic	11 (8)
Melanoma	7 (5)
Prostate	7 (5)
Renal	5 (4)
Cholangiocarcinoma	5 (4)
HCC	4 (3)
Carcinoid/neuroendocrine	4 (3)
Ovarian	4 (3)
Cervical	4 (3)
Unknown primary	3 (2)
Esophageal	3 (2)
Gastric	3 (2)
Sarcoma	3 (2)
CNS lymphoma	3 (2)
Endometrial	3 (2)
Other^b^	11 (8)
Malignancy was metastatic	72 (52)
**Receiving chemotherapy and/or immunotherapy**	
On active therapy	88 (64)
Therapy within last 3 months	10 (7)
Neutropenia (neutrophil count <500) at time of intervention	6 (4)
Infectious diseases consult during hospitalization	19 (14)
**Number of recommendations for each patient during study period**	
1 recommendation	114 (83)
2 recommendations	21 (15)
3 recommendations	3 (2)
30-day mortality^c^	37 (27)
90-day mortality^c^	60 (43)

IQR, inter-quartile range; SCC, Squamous cell carcinoma; HCC, hepatocellular carcinoma; CNS, central nervous system; IPPAF, in-person prospective audit and feedback.

a Includes NSCLC, SCC, and lung adenocarcinoma.

b Other: thyroid (n = 1), vulva (n = 1), urothelial (n = 2), Merkel cell tumor (n = 1), bladder (n = 2), glioblastoma multiforme (n = 1), osteosarcoma (n = 1), astrocytoma (n = 1), testicular (n = 1).

c Mortality was measured from the time of IPPAF intervention.

### Clinical syndromes and interventions associated with IPPAF:

The most commonly encountered clinical scenario was patients empirically treated with antimicrobials who were deemed unlikely to have a bacterial infection during IPPAF (Table 2). There was also a high rate of intra-abdominal pathology overall (30/154, 19%), often related to intra-abdominal malignancy or metastases.

**Table 2.** Clinical syndromes encountered during in-person prospective audit and feedback

Types of clinical syndromes	Interventions (n = 154)
Empiric treatment for syndrome which was determined not to be infectious (diagnostic error/momentum)^a^	33 (21)
Pneumonia	25 (16)
Urinary tract infection	12 (8)
Bacteremia (uncomplicated)	12 (8)
Cholangitis/cholecystitis	12 (8)
Skin and soft tissue infection	7 (4)
Fever undefined	5 (3)
Intra-abdominal abscess	5 (3)
Febrile neutropenia syndrome	5 (3)
End-of-life antibiotics	4 (2)
Unclear clinical syndrome	4 (2)
Peritonitis	3 (2)
Perforated viscus	3 (2)
Empyema/infected pleural fluid	3 (2)
*Pneumocystis jiroveci* prophylaxis dosing	2 (1)
Other^b^	19 (12)

a Included patients being presumptively treated for pneumonia (n = 10), spontaneous bacterial peritonitis (n = 5), UTI (n = 14), bacteremia (n = 1), infected pleural fluid (n = 1), cholangitis (n = 1), and prostatitis (n = 1).

b Other included C.difficile (n = 2), colitis not otherwise specified (n = 1), SBO (n = 2), long-term antibiotic prophylaxis (n = 2), failure of skin flap (n = 1), septic arthritis (n = 1), gut pneumatosis (n = 1), PJP (n = 1), short term prophylaxis (n = 2), abscess and wet gangrene (n = 1), cavitary lung lesion with neck mass (n = 1), cutaneous Candidiasis (n = 1), esophageal Candidiasis (n = 1), osteomyelitis (n = 1), oral HSV (n = 1).

Individual recommendations given in person over 34 weeks are shown in Table 3. Of the 154 interventions, 129 included specific patient care recommendations given in person and 25 additional patient cases were discussed without clear recommendations able to be made. Progress notes were documented in the chart for 31/154 (20%) interventions. Recommendations about appropriate duration were the most common intervention (63/154, 41%), followed by recommendations to stop antimicrobials in cases of diagnostic uncertainty (29/154, 19%).

**Table 3.** Types of in-person prospective audit and feedback interventions and acceptance rate

Types of interventions	Number of cases (n = 154)^a^	Stewardship note left in chart (% of cases)	Number of recommendations accepted (% of cases)
Duration	63	10 (16)	56 (89)
Stop antibiotics as syndrome is not clearly infectious (diagnostic uncertainty)	29	9 (31)	16 (55)
Clinical case discussion only^b^	25	0	NA
Stop antibiotics since fully treated	12	2 (17)	11 (92)
Narrow antibiotics	12	1 (8)	7 (58)
Conversion of intravenous to oral antibiotic therapy	12	4 (33)	9 (75)
Infectious diseases consult	4	4 (100)	3 (75)
Clarify diagnosis or workup	6	1 (17)	5 (83)
*Pneumocystis jirovecii* prophylaxis dosing	2	0	1 (50)

a Numbers exceed 154 because multiple recommendations were counted separately.

b In these cases, there was no stewardship recommendation, but complex cases were discussed with the medical team along with educational points. Examples include: the patient was recently admitted and the diagnosis was unclear, the patient was on antibiotics awaiting a diagnostic procedure, or the diagnosis (and whether it was infectious or not) was unclear even after workup.

### Hybrid PAF

Over the study period, 59 additional interventions for 58 patients were discussed outside of IPPAF, with recommendations made in person with one team member, via secure message to team pharmacist, or over the phone; progress notes were left for 23/59 (39%) of interventions performed. Clinical syndromes reflected in these interventions included antimicrobials given for bacteremia in 14/59 (24%), intra-abdominal infections in 11/59 (19%), pneumonia in 10/59 (17%), fever syndromes (neutropenic or non-neutropenic fever of unclear source) in 5/59 (8%), urinary tract infection (UTI) in 5/59 (8%), and asymptomatic bacteriuria in 5/59 (8%). Stopping antimicrobials or decreasing the duration was recommended in 24/59 (41%), changing antimicrobial choice (including IV to PO antimicrobials) was recommended in 20/59 (34%), and ID consult was recommended in 16/59 (27%) of cases.

### Feasibility of rounding three times weekly

Both physician and pharmacist were able to conduct IPPAF rounds on 42/102 (41%) potential days and at least one day on 21/34 (62%) weeks. Including weeks where hybrid PAF was performed, recommendations were given on at least one day 33/34 weeks (97%).

### Impact of IPPAF

**Figure 1.**
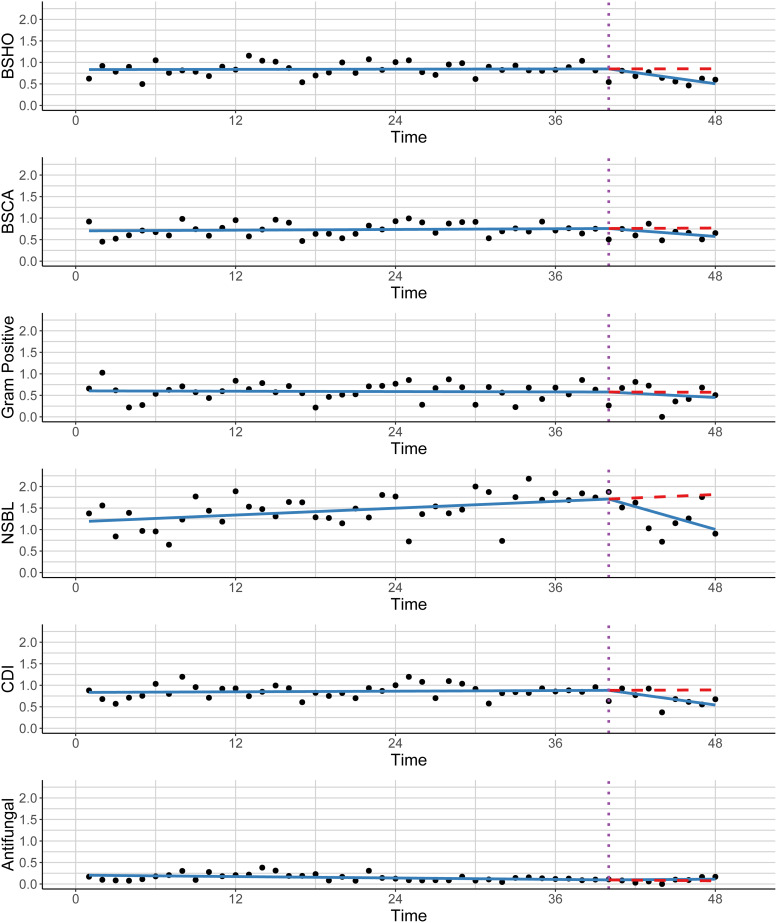
Standardized antimicrobial administration ratios (SAARs) for an oncology floor between 2020 and 2024. Dots represent the observed SAARs over time in months, the blue lines represent the predicted SAARs over time from the regression models, the start of IPPAF is marked by the vertical dashed purple line, and the red dashed line shows the counterfactual prediction, illustrating what the predicted SAARs would have been if the pre-intervention trends had continued during the intervention period. Each SAAR is included as its own trend: (A) Broad spectrum antibacterial agents predominantly used for community-acquired infections (“BSHO”) [cefaclor, cefdinir, cefixime, cefotaxime, cefpodoxime, cefprozil, ceftriaxone, cefuroxime, ciprofloxacin, ertapenem, gemifloxacin, levofloxacin, moxifloxacin]; (B) Broad spectrum antimicrobial agents predominantly used for hospital onset infections (“BSCA”) [IV amikacin, aztreonam, cefepime, ceftazidime, doripenem, IV gentamicin, imipenem/cilastatin, meropenem, piperacillin/tazobactam, IV tobramycin]; (C) Antibacterial agents predominantly used for resistant Gram positive infections (“Gram positive”) [ceftaroline, dalbavancin, daptomycin, linezolid, oritavancin, quinupristin/dalfopristin, tedizolid, telavancin, IV vancomycin]; (D) Narrow-spectrum beta lactam agents (“NSBL”) [amoxicillin, amoxicillin/clavulanate, ampicillin, ampicillin/sulbactam, cefadroxil, cefazolin, cefotetan, cefoxitin, cephalexin, dicloxacillin, nafcillin, oxacillin, penicillin G, penicillin V]; (E) Antibacterial agents posing highest risk for C.difficile infection (“CDI”) [cefdinir, cefepime, cefixime, cefotaxime, cefpodoxime, ceftazidime, ceftriaxone, ciprofloxacin, clindamycin, gemifloxacin, levofloxacin, moxifloxacin]; (F) Antifungal agents predominantly used for invasive candidiasis (“Antifungal”) [anidulafungin, caspofungin, fluconazole, micafungin].

There was a high rate of acceptance from clinical teams for IPPAF recommendations (108/129, 84%, excluding cases where only discussion occurred) (Table 3). The SAARs for our primary oncology unit over time are shown in Figure 1. The blue lines represent the predicted SAARs over time from the ITS models. The start of IPPAF is marked by the vertical dashed purple line. The red dashed line shows the counterfactual prediction, illustrating what the predicted SAARs would have been if the pre-intervention trends had continued during the intervention period. The slopes for monthly changes in the SAAR levels in the pre vs. post-IPPAF periods are shown in Table 4. The slopes for monthly changes in the SAAR levels in the pre vs. post-IPPAF periods are shown in Table 4. SAARs were generally stable in the pre-IPPAF period (NSBL and antifungal SAARs were increasing and decreasing, respectively), and they generally decreased during the post-intervention period. We detected statistically significant changes in slope for BSHO (*P* = .002), NSBL (*P* = .002), and CDI (*P* = .004) SAARs, with all three also significantly decreasing in the post-intervention period (*P* = 0.003, *P* = .004, *P* = .003, respectively).

**Table 4.** Changes in SAARs over time before and after IPPAF implementation. For each SAAR type, we provided estimates of the pre-intervention change over time (Pre-IPPAF Slope), the post-intervention change over time (Post-IPPAF Slope), and whether the pre-intervention and post-intervention slopes were significantly different from each other

SAAR Type	Pre-IPPAF Slope	**P** ^a^	Post-IPPAF Slope	**P** ^a^	**P diff** ^b^
Broad spectrum antimicrobial agents predominantly used for hospital onset infections (BSHO)	0.0004 (–0.004, 0.004)	0.84	–0.04 (–0.07, –0.02)	0.003	0.002
Broad spectrum antibacterial agents predominantly used for community-acquired infections (BSCA)	0.001 (–0.003, 0.005)	0.49	–0.02 (–0.05, 0.01)	0.09	0.09
Antibacterial agents predominantly used for resistant Gram positive infections (Gram positive)	–0.001 (–0.01, 0.005)	0.81	–0.02 (–0.07, 0.02)	0.39	0.44
Narrow-spectrum beta lactam agents (NSBL)	0.01 (0.004, 0.02)	0.004	–0.09 (–0.17, –0.02)	0.004	0.002
Antibacterial agents posing highest risk for C.difficile infection (CDI)	0.001 (–0.003, 0.01)	0.55	–0.04 (–0.07, –0.01)	0.003	0.004
Antifungal agents predominantly used for invasive candidiasis (Antifungal)	–0.003 (–0.005, –0.001)	0.01	0 (–0.01, 0.02	0.74	0.48

SAAR, Standardized Antimicrobial Administration Ratio; IPPAF, In-person prospective audit and feedback.

a P represents *P* values from tests determining if the estimate of the slope is equal to zero or not.

b P diff represents the *P* value for a test determining whether there are differences in pre- and post-IPPAF slopes.

## Discussion

We present the first description of prospective audit and feedback focused on an inpatient cohort with oncologic malignancies. This 34-month IPPAF program was feasible, identified several clinical syndromes unique to this patient population, resulted in a high rate of acceptance of stewardship recommendations, and was associated with a statistically significant decrease in several SAARs on a ward that housed oncology patients.

Although the ASP team was only able to conduct IPPAF rounds on 41% of potential days and 62% of potential weeks, continuity was maintained by hybrid PAF, resulting in ASP discussions held 97% of weeks in our study period. Feasibility and time for IPPAF should be considered when analyzing how much FTE is required for ASPs^11^.

Among our patients, the most commonly observed clinical syndromes were consistent with previous studies of inpatient PAF and included pneumonia, UTI, and bacteremia^12^. However, our oncology patients also had a notably higher rate of intra-abdominal syndromes including cholangitis, intra-abdominal abscess, and perforated viscus (Table 2). Many of these infections occurred as a direct result of the location of the malignancy. Other clinical syndromes specific to our patients included febrile neutropenia syndromes such as neutropenic enterocolitis, infected pleural effusions (with or without pleural catheters), and end-of-life antimicrobials. There are some data that inform best clinical practices in these scenarios^13,14^, but not for others (neutropenic enterocolitis, infected pleural effusions). End-of-life antimicrobials are often justified for patient comfort, but there are limited data to guide which patients benefit^15–17^.

To our knowledge, this is the first study of IPPAF with a focus on AU in patients with oncologic malignancies. The rate of acceptance of recommendations in our oncology patients was high, which is consistent with prior studies examining IPPAF in a variety of clinical settings^18–23^. The acceptance rate was higher for recommendations that involved modification of duration or antimicrobial agent rather than for recommendations to stop antimicrobials because the syndrome did not warrant them. This finding is also consistent with previous studies of PAF^12,19,24^, and may reflect clinicians’ perception that the benefit of “just in case” antimicrobials is higher than the risks, particularly in this patient population^8,19,25^.

Stopping antimicrobials in cases of diagnostic uncertainty—that is, cases in which patients were empirically treated for an infectious syndrome but where there was little or no evidence—was a common intervention. These cases included treatment given for asymptomatic bacteriuria, respiratory signs or symptoms misdiagnosed as pneumonia, and notably peritonitis, which provided opportunities for education. For example, we observed that teams were sampling peritoneal fluid among patients with malignancy-related ascites, and then prescribing antimicrobials to patients with ascitic polymorphonuclear leukocyte (PMN) count >250. However, while PMN-guided treatment of peritonitis is recommended among patients with cirrhosis^26^, this threshold has not been validated among patients with malignancy-related ascites, and PMN counts are frequently high even among uninfected patients with malignant ascites^27,28^. Our observation provided an opportunity for targeted education. However, the rate of antimicrobial cessation among cases with diagnostic uncertainty was lower than other recommended interventions (Table 3), suggesting that interventions optimizing diagnostic test ordering may be superior^29^.

Harms of antimicrobials are well-demonstrated in the general population^30,31^, and, specific to the oncology population, the impact of AU on the microbiome is hypothesized to be linked to malignancy outcomes^32–34^; addition of IPPAF, if feasible, may be a beneficial component of immunocompromised ASPs. The high observed mortality rate in our cohort was notable for a few reasons (Table 1). First, as demonstrated in our study and others, oncology patients receive many antimicrobials, especially broad-spectrum antimicrobials^35^, near the end of life. Inpatient oncology ASPs should recommend antimicrobials that are least likely to cause adverse effects or toxicity and be given for the shortest effective duration focused on symptom alleviation^15–17,25,36,37^. Second, one of the major goals of ASPs is to decrease population-level resistance. Antibiotic overuse is hypothesized to contribute to population-level resistance through inter-personal transmission of antibiotic-resistant colonizers^38^. Since our patients did not live long following their

inpatient stay, it is unclear whether their subsequent exposure to the community or hospital environment would be sufficient to contribute to population-level resistance. As antibiotic use increases, these questions are important to explore to appropriately distribute limited ASP resources. A larger implementation study should investigate programs such as the one described here to fully assess the impact of IPPAF on prescribing, antimicrobial resistance, and other patient safety outcomes.

The SAARs for the primary unit on which our oncology patients resided generally decreased, with significant decreases in our BSHO, NSBL, and CDI SAARs during our IPPAF period, suggesting a decrease in AU relative to similar units at other institutions. We examined the impact of IPPAF on antibiotic prescribing using SAAR data rather than AU data so that we could more completely assess changes in larger categories of AU versus individual agents. Additionally, examining SAAR data allowed us to better understand how our hospital performs compared to similar institutions. The SAAR is an AU metric created to help benchmark categories of AU across institutions; however, published examples of ASPs using the SAAR to track and measure impact of interventions aimed at improving prescribing are limited. We hope that our analysis adds to the literature by providing an example of how ASPs can use the SAAR to track the impact of their interventions.

Our study has significant limitations. We included data from a single center which limited sample size. We prospectively documented patients on antimicrobials but did not track all patients on each admitting service, thus we were unable to describe whether service-level (rather than unit-level) AU changed over time. In addition, since our oncology ward also admitted patients with non-oncologic malignancies, the impact of IPPAF on unit-level SAARs may be diluted due to a mixed patient population. Due to competing clinical obligations, we were unable to conduct IPPAF rounds consistently three times per week during the intervention; however, this is similar to a real-world ASP practice with multiple competing obligations. We were unable to quantify whether inability to consistently perform IPPAF three times weekly resulted in missed opportunities. Our ASP has dedicated resources for an oncology-focused IPPAF program whereas many programs do not; therefore, this may limit generalizability of our data to other ASPs. However, since hospital ASPs are expected to cover all inpatients, our data also support increasing stewardship resources in order to adequately address all populations.

In conclusion, we describe the development and impact of an immunocompromised ASP with a focus on an inpatient oncology population^3,39,40^. We identified numerous ASP opportunities. Our IPPAF program was associated with a high rate of acceptance of ASP recommendations and lower SAARs during the intervention period, suggesting likely benefit of IPPAF among other high-risk patient populations at our institution and elsewhere. Although immunocompromised patients are often excluded from studies evaluating AU, which can preclude major ASP efforts in this population, our study demonstrated numerous ASP opportunities and high acceptance of IPPAF recommendations. Our experience provides insights and lays the groundwork for other ASPs as they expand to immunocompromised populations.

## Supporting information

Imlay et al. supplementary material Imlay et al. supplementary material

## Supplementary material

For supplementary material accompanying this paper visit http://doi.org/10.1017/ash.2024.446. click here to view supplementary material
