# Supplementary material for: In-person prospective audit and feedback on an oncology ward: development of an immunocompromised antimicrobial stewardship program
Source: Antimicrob Steward Healthc Epidemiol. 2024 Oct 15;4(1):e173. doi: 10.1017/ash.2024.446 (PMC11488469; doi:10.1017/ash.2024.446)

**Supplemental Figure 1AB.** Flowsheet template in Epic for leaving stewardship progress notes (A). Formal notes additionally include a case summary and summary of data; an example of data summary for Gram negative bacteremia shown in (B).
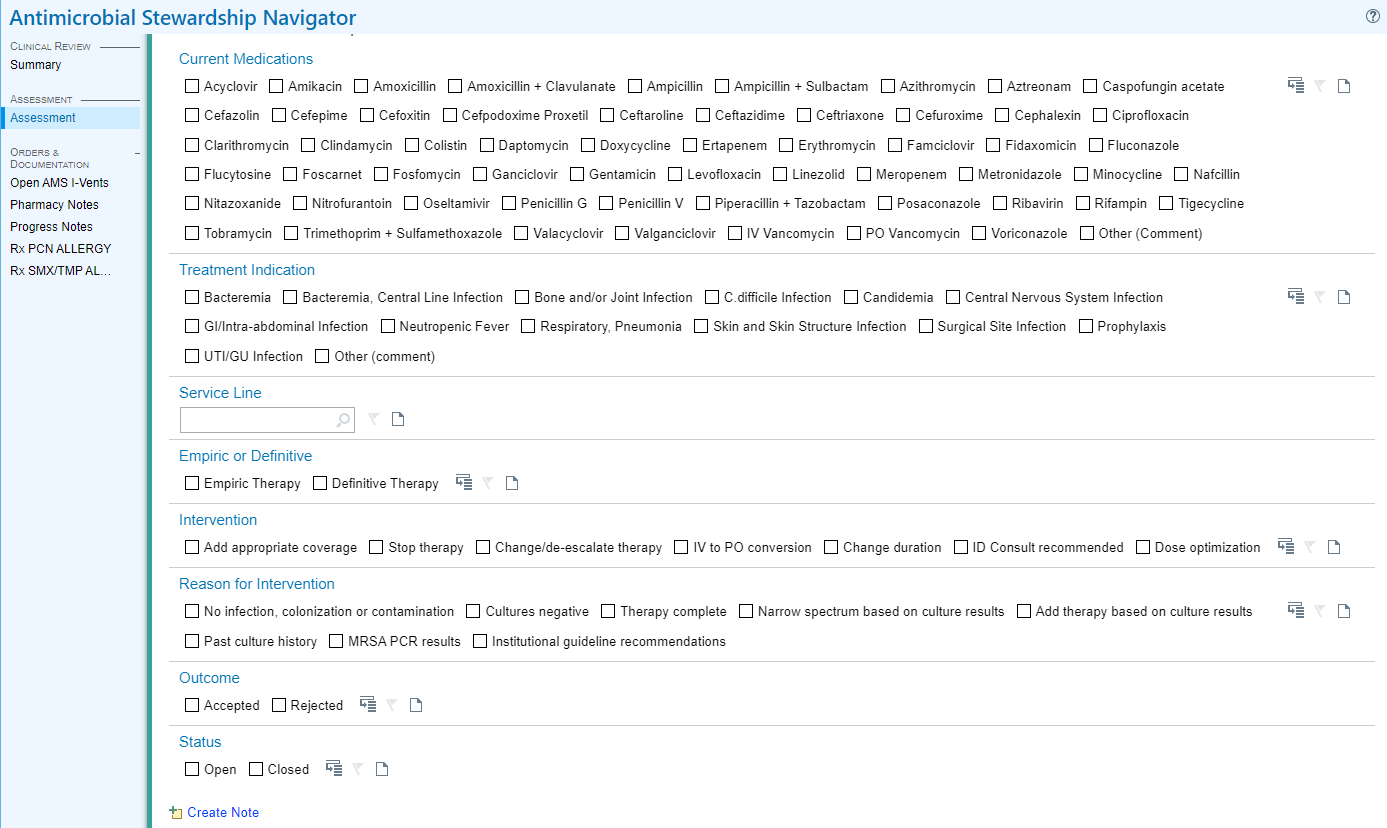


B.


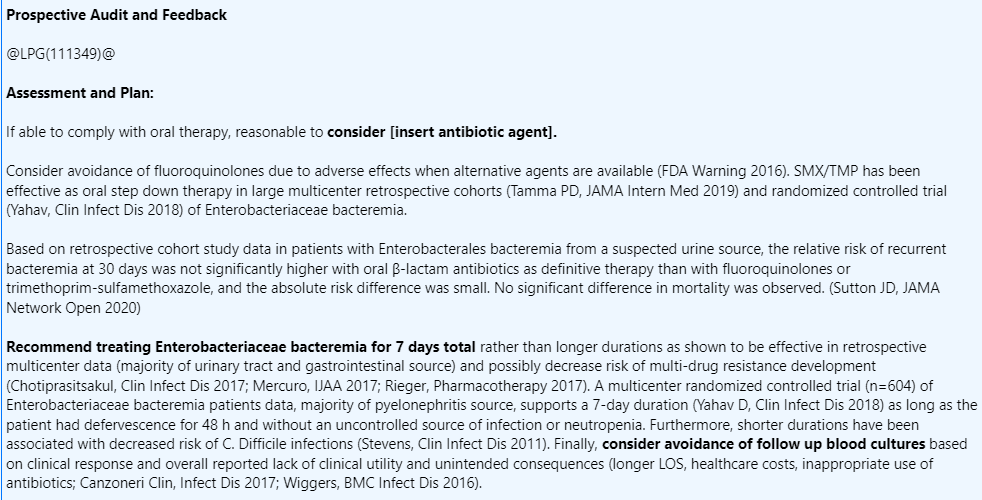

Supplement: Imlay et al. supplementary material [file S2732494X24004467sup001.docx]
